# Environmental risk factors for multiple sclerosis: a comprehensive systematic review and meta-analysis

**DOI:** 10.1007/s00415-025-13248-0

**Published:** 2025-07-15

**Authors:** Bruno Kusznir Vitturi, Maria Cellerino, Daniele Boccia, Emmanuelle Leray, Jorge Correale, Ruth Dobson, Ingrid van der Mei, Kazuo Fujihara, Matilde Inglese

**Affiliations:** 1 Department of Neurology, University Hospital Schleswig-Holstein, Campus Kiel and Kiel University, Arnold-Heller-Str 3, 24105 Kiel, Germany; 2 Department of Neuroscience, Rehabilitation, Ophthalmology, Genetics, and Mother-Child Health (DINOGMI), University of Genoa, Genoa, Italy; 3 UMR 6051, EHESP, CNRS, Inserm, ARENES, RSMS U 1309, Rennes University, Rennes, France; 4 Department of Neurology, of Biological Chemistry and Biophysics, Fleni, Institute, CONICET/University of Buenos Aires, Buenos Aires, Argentina; 5 Centre for Preventive Neurology, Wolfson Institute of Population Health, Queen Mary University of London, London, EC1M 6BQ UK; 6 Menzies Institute of Medical Research, University of Tasmania, 17 Liverpool Street, Hobart, TAS 7000 Australia; 7 Department of Multiple Sclerosis Therapeutics, School of Medicine and Multiple Sclerosis and Neuromyelitis Optica Center, Fukushima Medical University, Southern TOHOKU Research Institute for Neuroscience, Koriyama, Japan

**Keywords:** Demyelinating diseases, multiple sclerosis, environmental, risk factors, neuroepidemiology

## Abstract

**Background and Objectives:**

Multiple sclerosis (MS) is a demyelinating disease of the central nervous system. Its etiology may involve both genetic and environmental factors, including vitamin D levels, body mass index, infections, and smoking. This is the first comprehensive systematic review with meta-analysis that synthesizes and explore the role of many environmental risk factors in the etiology of MS.

**Methods:**

A systematic search of MEDLINE, SciVerse ScienceDirect and Web of Science were conducted for any original peer-reviewed article that included adult subjects diagnosed with and without MS that were exposed to any environmental risk factor. We did not set any time restrictions. Data were extracted and the quality assessment was performed with the Critical Appraisal Checklist for Case Control Studies. All the information was synthesized qualitatively and quantitatively (meta-analysis). We used the random-effects model based on the binomial distribution to calculate the pooled effects sizes (ES) regarding the risk of developing MS according to each potential risk factor.

**Results:**

One-hundred thirty-two publications met all the eligibility criteria and were included in the review. Overall, 109,626 people with MS and 16,724,390 controls from 38 countries were included in the review. A total of 42 environmental risk factors were investigated as potential risk factors for MS. Among the various statistically significant associations, the pooled ES revealed a direct association between serological evidence of contact with EBV (ES = 1.96, 95% CI = 1.53–2.51), herpes virus type 6 (HHV-6) (ES = 2.84, 95% CI = 2.08–3.89) and varicella-zoster virus (ES = 1.33, 95% CI = 1.08–1.63) and the occurrence of MS. Similarly, smoking was associated with a greater likelihood of having MS (ES = 1.43, 95% CI = 1.27–1.61). Vitamin D levels at any time were negatively associated with the proportion of cases of MS and had a moderate pooled ES (g = – 0.48, 95% CI = – 0.88–0.09). Adult BMI was not associated with MS.

**Discussion:**

This review furnishes a broad and detailed overview of the potential environmental risk factors associated with MS. Our findings hold notable implications for public health policies, clinical practices, and the focus of future research.

**Supplementary Information:**

The online version contains supplementary material available at 10.1007/s00415-025-13248-0.

## Introduction

Multiple sclerosis (MS) is the most common demyelinating disease of the Central Nervous System (CNS) affecting at least 2.8 millions of individuals worldwide [1]. Despite current knowledge about MS pathogenesis and increasing understanding around immune cell dysfunction resulting in demyelination, inflammation, and axonal damage, [2] there remain many unanswered questions about disease aetiology.

In high-risk countries MS incidence is estimated to be around 5–20 per 100,000 inhabitants with recent estimates highlighting a significant increase over recent decades [3]. MS is caused by the interplay of genetic and environmental factors. Large genome wide association studies have indicated the role of genetic predisposition in MS aetiology, in particular the expression of specific alleles in immune response regulatory genes [4]. However, this genetic component alone cannot completely explain MS pathogenesis, and in particular do not explain the rapid increase in incidence observed over recent decades [3].

A multitude of environmental factors have been proposed as potential contributors to the development of MS. Among these, vitamin D levels [5], body mass index (BMI) [6], infections including Epstein-Barr virus [7, 8], smoking [9] and gut microbiome [10] have emerged as prominent candidates. These factors, diverse in their nature and mechanism, collectively emphasize the need for a holistic approach when investigating MS aetiology. The investigation of environmental factors in MS is of paramount importance for several compelling reasons.

First it offers the opportunity to uncover key insights into the interplay between genes and the environment, shedding light on potential mechanisms that underlie MS development [11]. As genetic factors alone cannot account for the increasing incidence of MS, understanding how environmental factors interact with genetic predisposition is important. This knowledge can facilitate the identification of at-risk populations and inform targeted preventive strategies [12]. Secondly, some of these environmental factors are modifiable. For instance, lifestyle interventions that address factors such as BMI [13] and smoking habits [14] hold the potential to mitigate MS risk. By identifying and understanding these modifiable risk factors, actionable strategies to reduce the incidence and burden of MS can be developed [14]. Thirdly, an exploration of environmental factors may provide valuable insight into the mechanism underpinning MS pathogenesis [11]. Each factor offers a unique window into the complex interplay between the immune system, the nervous system, and the environment. As we delve deeper into the effects of these factors on MS susceptibility and progression, we may uncover new pathways and targets for therapeutic intervention.

Most reviews on environmental risk factors have been narrative reviews [15, 16]. To the best of our knowledge, there is no previous systematic review with meta-analysis that comprehensively addresses all the environmental factors potentially linked to MS and already described in the literature. In addition, previous meta-analyses of specific risk factors are out of date, as this topic is of growing interest and many original publications have been published recently. A systematic review with a quantitative approach may clarify the potential weight of multiple risk factors, alongside providing the highest degree of evidence in the field. This meta-analysis seeks to examine the current role these environmental risk factors may play in MS development. By synthesizing existing research on environmental risk factors, we aim to contribute to a deeper understanding of the multifaceted nature of MS aetiology.

## Methods

### Protocol and registration

The present systematic review was designed and performed following the Preferred Reporting Items for Systematic Reviews and Meta-analyses (PRISMA) [17] statement (eTable 1). The protocol was registered in the International Prospective Register of Systematic Reviews (PROSPERO) with the registration number CRD42023470960. It was not necessary to obtain approval from the local ethics committee or a written consent form due to the design of the study, which does not involve the direct recruitment of research subjects.

### Data sources and search strategy

A systematic literature search was carried out using three electronic academic databases—PubMed/MEDLINE, SciVerse ScienceDirect, Web of Science—between December 2023 and July 2024. The detailed search strategy is presented in eTable 2. The search results were exported and managed in Zotero (Corporation for Digital Scholarship—version 6.0.27).

### Study selection

After removing duplicate entries, titles and abstracts were examined to assess the potential relevance of the publications and remove those that did not fit the topic. For all the studies retrieved, two independent, previously trained researchers (BKV and MC) reviewed the full text to determine their eligibility according to the pre-established inclusion and exclusion criteria, each remaining blind to the other's decision. In the event of conflicting opinions, a senior researcher (MI) was consulted to promote discussion and reach a consensus.

The inclusion criteria were as follows. We included any original peer-reviewed publication that included adult patients diagnosed with MS and whose characteristics were compared with individuals who did not develop the disease. The diagnosis should be supported by the current standard guidelines in effect during the study period and/or confirmed by a neurologist. All research subjects needed to have recorded a measure of exposure to an environmental risk factor during adulthood. Finally, we only included studies with clear evidence that exposure to the risk factor occurred or started prior to the MS diagnosis, even if the measurement of the exposure was conducted after MS onset. This was the case when the measure allowed for inferences, such as serological evidence (IgG) of a previous infection combined with a past positive medical history, or when the variable was considered by the study authors to be stable over time (e.g., obesity).

Environmental variable risk factors were defined as modifiable, non-genetic elements in an individual's external context that can influence the likelihood of developing a disease or condition. Adulthood is defined as the stage that begins at 18 years of age and continues until death. The outcome of the studies was MS diagnosis, with studies reporting non-specific demyelinating events only not accepted. No time restrictions were applied to the search or inclusion of studies. Publications written in English, Spanish, French, Italian and Portuguese were accepted. Eligible studies included longitudinal case–control studies, nested case–control studies, cross-sectional studies, and ecological studies. Publications conceived as reviews, clinical trials, conference abstracts, letters to the editor, expert opinions, commentaries, case reports, case series and editorials were excluded. When more than one publication reported from the same cohort/data set and addressed the same variables, the most recent paper was selected, and the other(s) excluded. Studies that investigated the onset of MS in subjects with a preceding demyelinating event (e.g., optic neuritis) or that compared cases to controls diagnosed with other neurological diseases were excluded. We also excluded studies with mixed variables or characteristics — that is, environmental factors possibly involving an important intrinsic component as well, such as sleep disorders. We did not accept studies that evaluated a heterogeneous group of several specific variables as a potential risk factor (e.g., studies that evaluated"vaccination"in general, and not a specific vaccine). Some preliminary studies have assessed the risk of detailed nutritional profiles and the risk of MS. These studies were not included in this review due to the inability to identify a clear and objective dietary profile that could be grouped with other dietary profiles and studied in a meta-analysis. Studies specifically addressing risk factors during childhood in the context of pediatric multiple sclerosis were beyond the scope of this review and were excluded. This decision was based on the distinct characteristics of pediatric MS compared to its manifestation in adults.

Exposure to any biological agent was defined as serological evidence of contact with the pathogen. Vaccination status was accepted if self-reported or documented in any vaccination record or certificate. Tobacco exposure was classified as either active smoking or passive smoking, defined as the inhalation of tobacco smoke by individuals other than the smoker. Vitamin D levels were classified based on serum concentrations of 25-hydroxyvitamin D, following the Endocrine Society's recommendations [18]. Deficiency was defined as levels below 20 ng/mL (50 nmol/L), while insufficiency ranged from 20 to 29 ng/mL (50–74 nmol/L). Sufficient levels were between 30 and 100 ng/mL (75–250 nmol/L).

BMI was used to classify weight status, adhering to the World Health Organization (WHO) guidelines [19]. A BMI between 18.5 and 24.9 was classified as normal weight, representing a healthy range. Overweight was defined as a BMI between 25 and 29.9, while obesity was categorized as a BMI of 30.0 or higher. For the last two categories, the number of participants classified in each subcategory was compared with the remaining number of study participants. Exposure to other environmental risk factors cited in the literature was considered valid if self-reported by the research participant for any time prior to the MS diagnosis.

### Data extraction

In addition to the number of cases and controls with MS and the number of exposed and non-exposed participants in each cohort, data on the first author, year of publication, country, sample size, mean age, gender, mean disease duration and mean Expanded Disability Status Scale (EDSS) history were extracted and recorded. For publications lacking data on the number of exposed and non-exposed individuals or cases and controls, we contacted the corresponding author via email to request additional information. Whenever our contact attempt failed, the study was excluded from the analysis. When a multicenter study provided results for each country, the data were separated and analyzed individually. For multicenter studies without detailed discrimination of cohorts by country, the entire sample was considered as a whole. All extracted data were double checked one month after the initial extraction to optimize reliability and minimize the risk of bias. The quality assessment was performed with the Critical Appraisal Checklist for Case Control Studies, developed, and validated by the Joanna Briggs Institute. It comprises ten questions for which researchers can answer ‘yes’, ‘no’, ‘unclear’ or ‘not applicable (NA)’ in response to each item. The greater the number of ‘no’ or ‘unclear’ selected, the greater the risk of bias in each category and in each study. The critical appraisal was carried out considering the variables of interest in our review. This step was also carried out by two independent and previously trained researchers, always considering the opinion of a third researcher in case of discrepancy.

### Statistical analysis

All the information was synthesized qualitatively and quantitatively (meta-analysis). The environmental risk factors found in the literature were grouped according to the main thematic area and the number of studies available for each risk factor. Based on the way the studies included in the review reported the information, all the risk factors were considered as categorical variables. We used the random-effects model based on the binomial distribution to calculate the pooled effects sizes regarding the risk of developing MS according to each potential risk factor. Effect sizes (ES) were calculated in log odd ratios and were then exponentiated to be reported. Vitamin D levels were also analyzed as numerical variables and, in this case, ES was measured using Hedges'g. Studies were excluded from the analysis if they used varying cut-off points for environmental variables, making it impossible to group them quantitatively. For example, a study that assessed BMI by asking participants to identify themselves in images of people with different body compositions, while all the other studies calculated BMI precisely. Or a study that classified vitamin D deficiency without mentioning the cutoff point or using one not supported by any guideline. Potential influences on pooled estimates were investigated using subgroup analyses and meta-regression. The latter was only carried out only when there were more than 10 studies in the analysis and considered all the independent variables. Between-study heterogeneity was assessed using the I^2^ statistic and visually inspecting the forest plot. We investigated the existence of publication bias using Egger’s linear regression test and with the visual inspection of the funnel plots. A p < 0.05 was considered statistically significant. All statistical analyses were performed using STATA/BE 17.0.

## Results

**Fig. 1:**
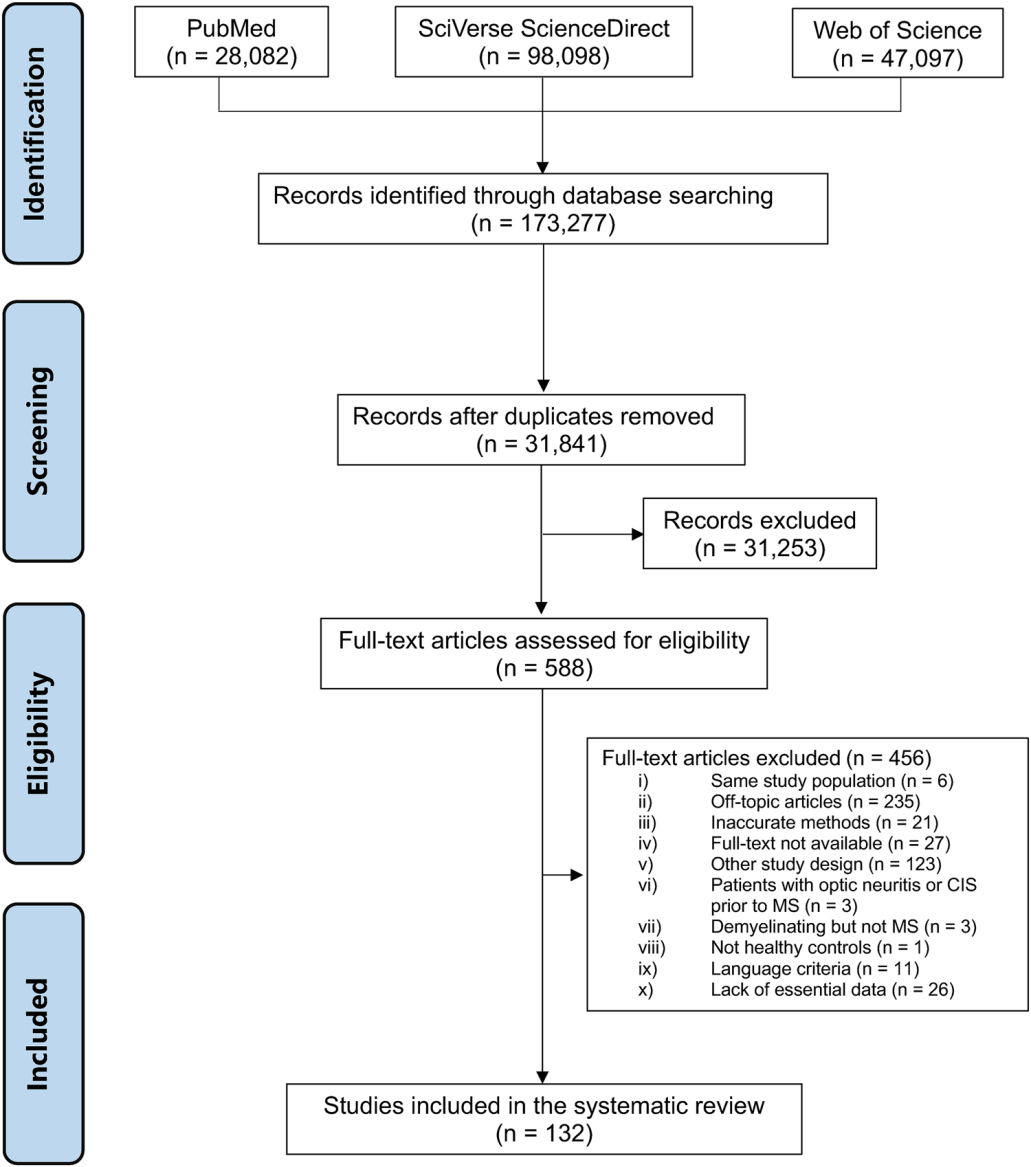
PRISMA flowchart

A total of 173,277 publications matched the search terms. After removing the duplicates, 31,841 publications were screened by reading the title, abstract, and full text. Finally, 132 publications met all the eligibility criteria and were included in the review (Fig. 1). The studies were carried out in 38 countries from 6 continents: Australia, Canada, China, Cuba, Cyprus, Denmark, Finland, France, Germany, Hungary, Iceland, India, Indonesia, Iran, Ireland, Italy, Japan, Jordania, Kuwait, Lebanon, Mexico, Morocco, Norway, Poland, Portugal, Russia, Saudi Arabia, Serbia, Serbia, South Africa, Spain, Sweden, Taiwan, The Netherlands, Turkey, The United Kingdom, and The United States of America. The median year of publication of the studies was 2015 (range: 1969–2023). The studies are fully described in eTable 3.

Overall, 104,320 people with MS (PwMS) and 16,714,698 controls were included in the analysis. The average age of PwMS varied from 23.4 to 52.6 years, and the proportion of women ranged from 24.3 to 100.0%. The quality of most publications was satisfactory (eTable 4). A reduction in quality was most frequently observed due to not assessing the possible confounding factors (Questions 6 and 7) and duration of exposure (Question 9). In this review, a total of 42 environmental risk factors were investigated as risk factors for MS.

### Infections

**Fig. 2:**
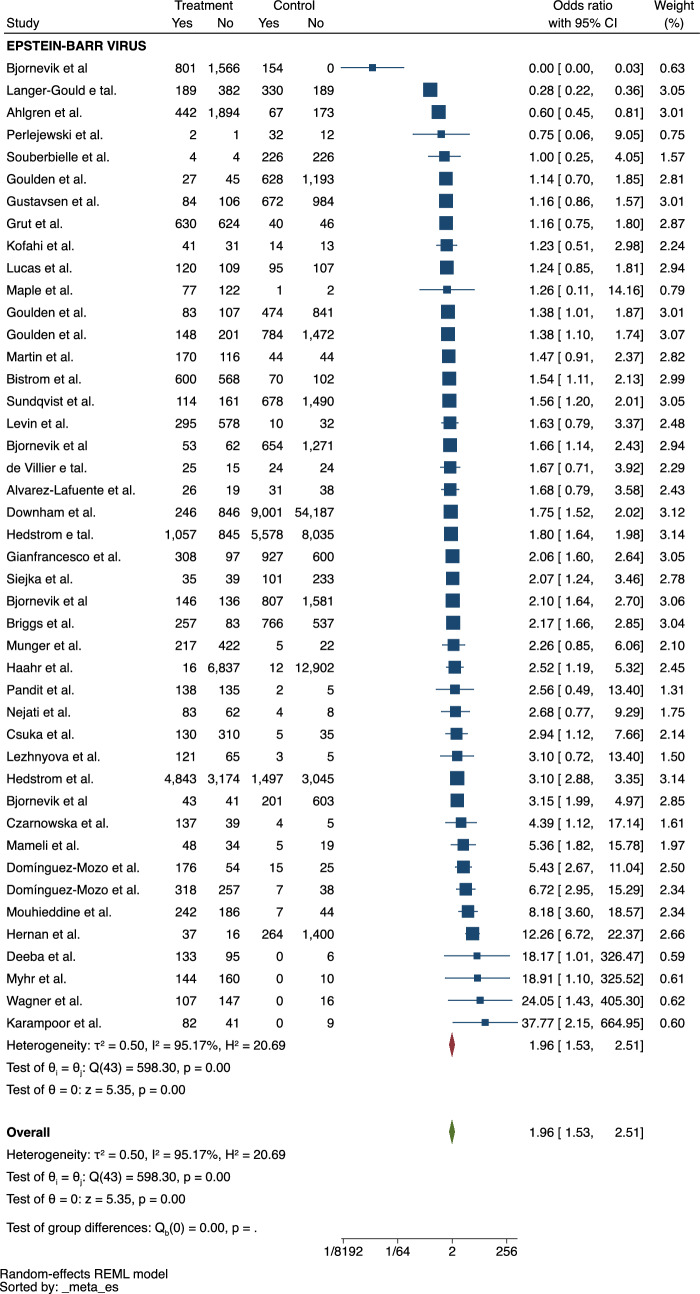
Meta-analysis of the association between EBV and MS

There were 44 studies included in the review that investigated the role of previous EBV infection on the risk of MS (Fig. 2). This outcome was assessed through EBV serology, which was performed using indirect immunofluorescence (VCA) or anticomplement immunofluorescence (EBNA complex, EBNA-1, EBNA-2) to detect IgG antibodies. The pooled ES revealed a positive association between EBV infection and the occurrence of MS (ES = 1.96, 95% CI = 1.53–2.51). There was no publication bias in this analysis.

**Fig. 3:**
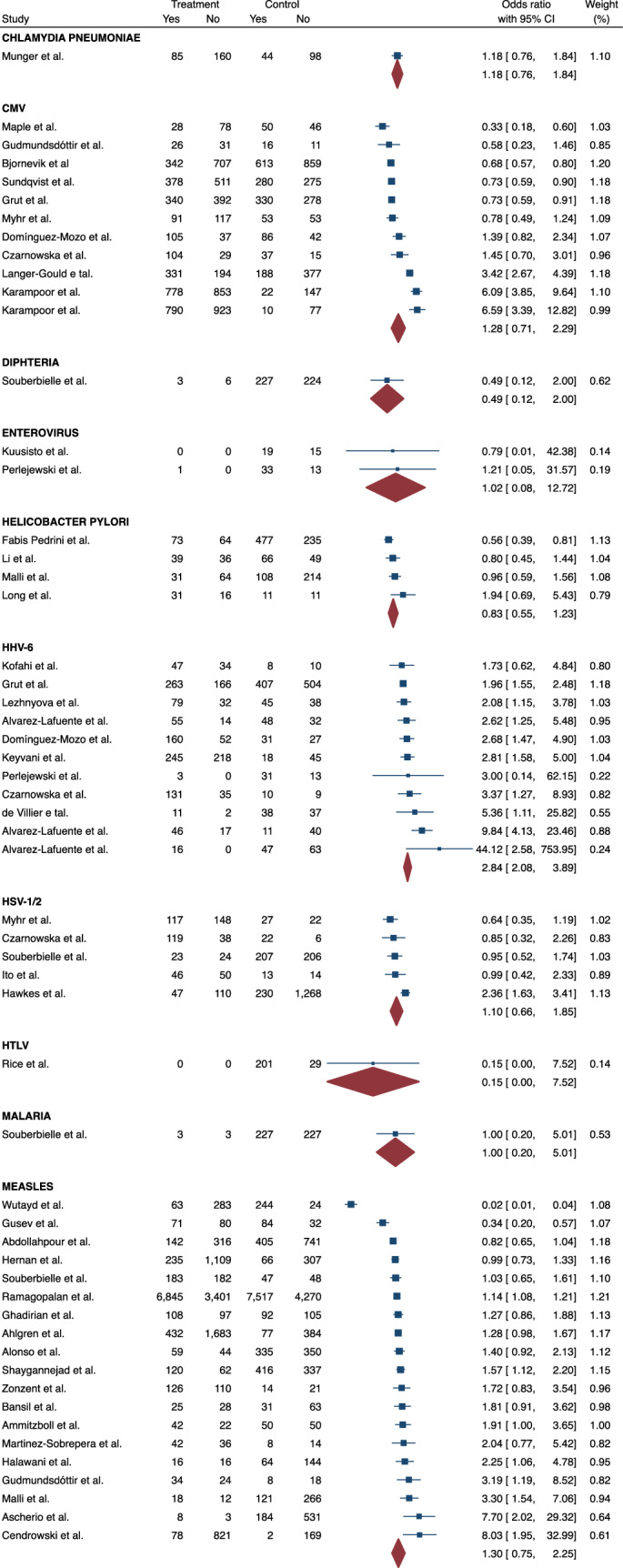

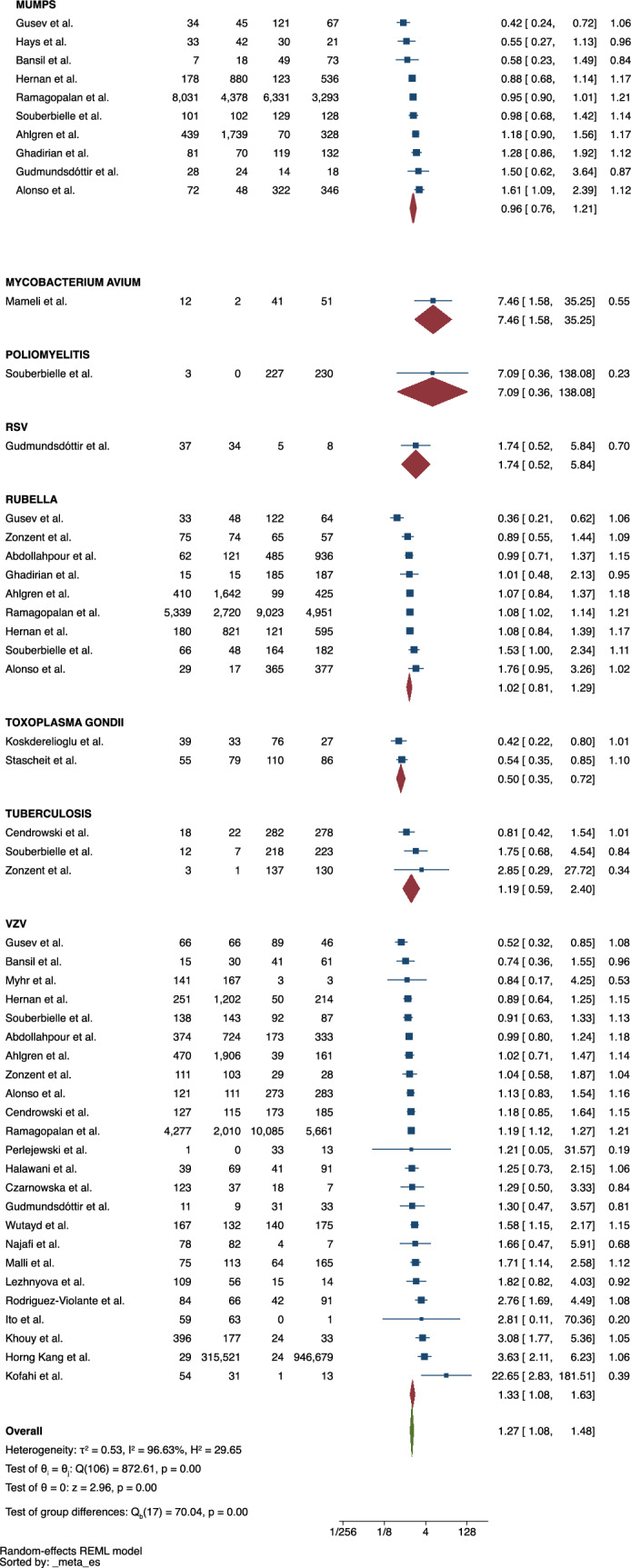
Meta-analysis of the association between infections and MS

The possible influence of other 18 different infections on subsequent risk of MS were additionally investigated, 11 of which were caused by viruses, 5 by bacteria and 2 by protozoa (Fig. 3). Infections with herpes virus type 6 (HHV-6) and varicella-zoster virus (VZV) were associated with a greater likelihood of having MS (ES = 2.84, 95% CI = 2.08–3.89 and ES = 1.33, 95% CI = 1.08–1.63, respectively). There was no statistically significant association for any other pathogen. The heterogeneity of the studies was high (I^2^ = 95.179) and a publication bias was detected (p = 0.009).

### Vaccines

Nine vaccines have been studied as potential risk factors for MS (eFigure 1). Combining all studies, irrespective of immunization type, immunization was associated with MS diagnosis (ES = 1.73, 95% CI = 1.12–2.67). Among all immunization types, only a tetanus immunization prior to onset was associated with MS (ES = 4.15 (95% CI = 2.89–5.96), but this data came from a single study. No other vaccine has been associated in any way with MS. The heterogeneity of the studies was high (*I*^*2*^ = 99.50%) and there was a significant degree of publication bias (*p* < 0.001).

### Smoking

**Fig. 4:**
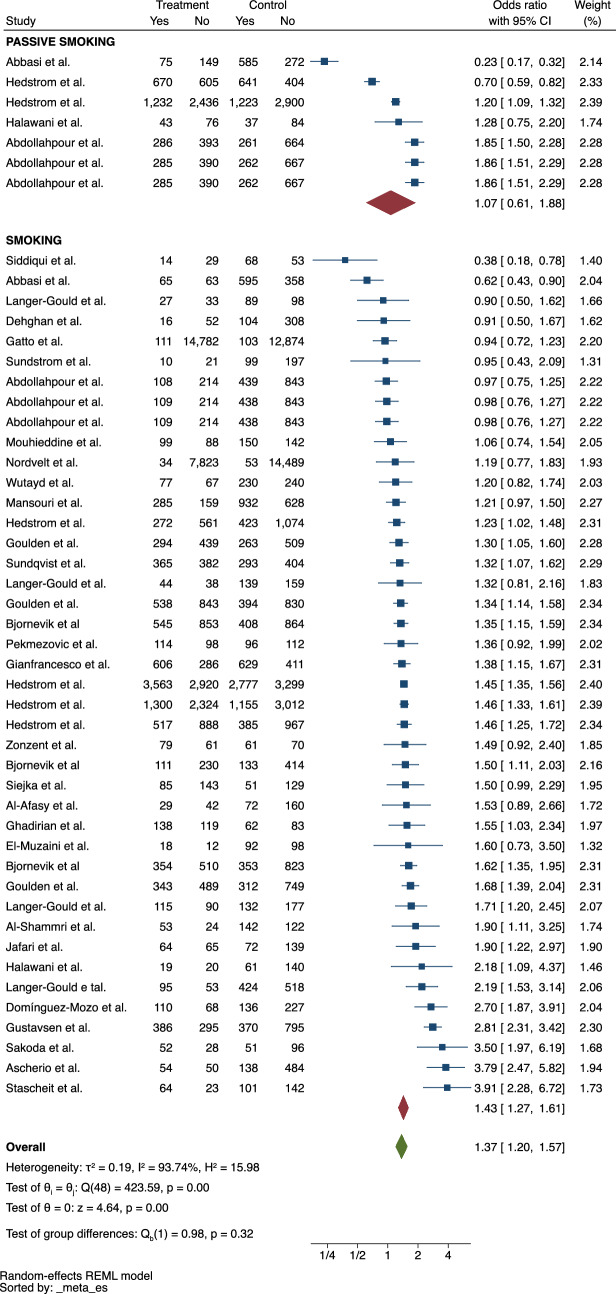
Meta-analysis of the association between smoking and MS

Smoking was associated with a greater likelihood of having MS (ES = 1.43, 95% CI = 1.27–1.61) (Fig. 4). Studies included both current smokers and former smokers. No statistically significant association was observed in the case of passive smoking. The heterogeneity of the studies was significant (*I*^*2*^ = 93.74%). No publication bias was identified (*p* = 0.84).

### Body mass index (BMI)

The studies available in the literature compared participants classified within each of the body composition categories (normal weight, overweight, obesity) with other subjects belonging to different categories. The BMI was not associated with the proportion of cases of MS (ES = 0.94, 95% CI = 0.85–1.04)—patients with normal weight, overweight and obesity had similar effect sizes (eFigure 2). There was no significant heterogeneity among the studies (*I*^*2*^ = 27.73%). There was publication bias for this analysis (*p* < 0.01).

### Vitamin D

**Fig. 5:**
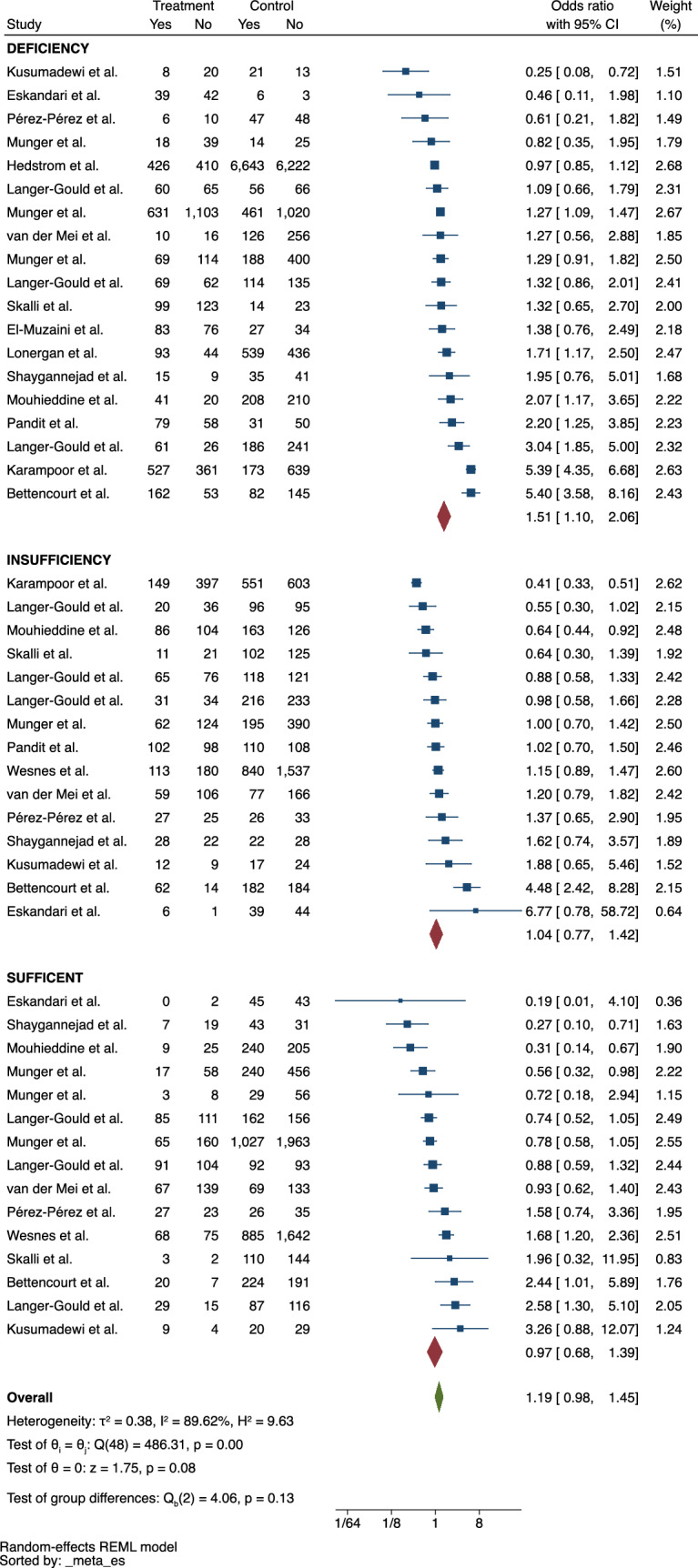
Meta-analysis of the association between vitamin D status and MS

Vitamin D deficiency was associated with the occurrence of MS (ES = 1.51, 95% CI = 1.10–2.06) (Fig. 5). Studies assessed vitamin D levels at different time points prior to diagnosis, and most did not specify them. Meta-regression revealed that no other variable was associated with the outcome. When measured as a continuous variable, vitamin D levels were negatively associated with the proportion of cases of MS and had a moderate pooled ES (g = – 0.48 ng/mL, 95% CI = – 0.88 ng/mL,− 0.09 ng/mL) (eFigure 3). The heterogeneity of the studies was not insignificant in both analyses (I^2^ = 89.62% and I^2^ = 98.42%). No publication bias was identified (*p* = 0.59 and *p* = 0.32).

### Other environmental risk factors

**Fig. 6:**
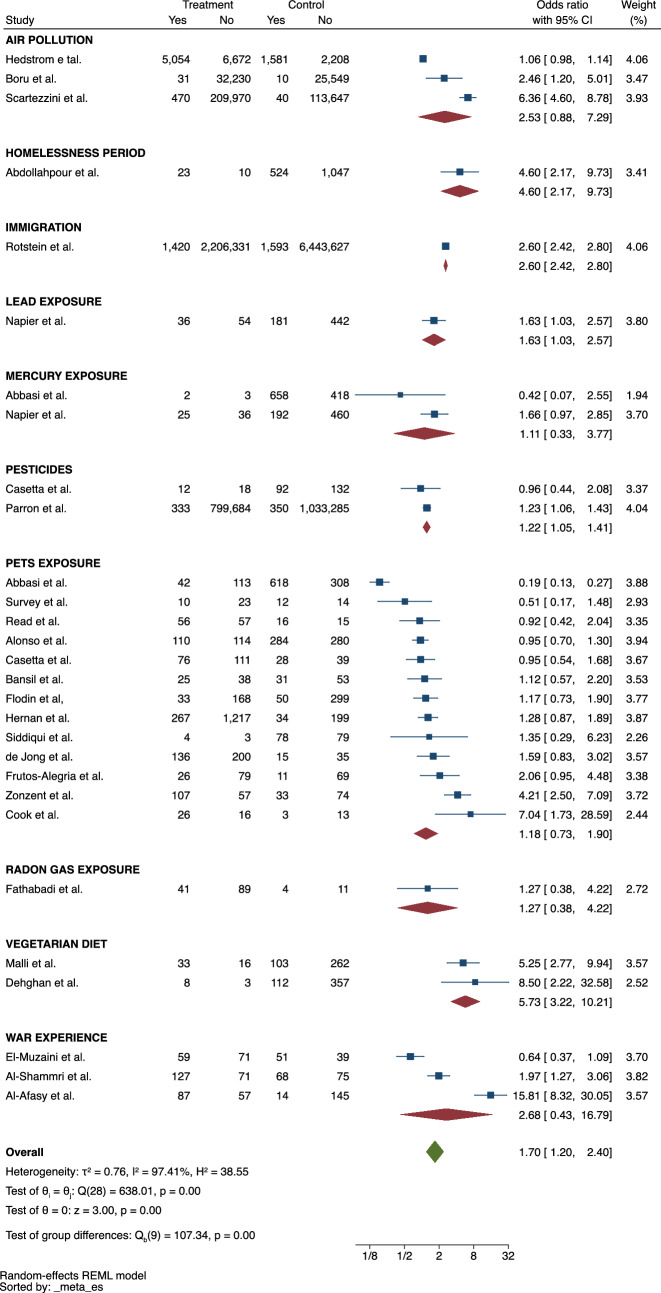
Meta-analysis of the association between other environmental risk factors and MS

There were 10 other potential environmental risk factors described in the literature (Fig. 6). Having a vegetarian diet was associated with a 5 to 6 times higher risk of developing MS compared to not having a vegetarian diet (ES = 5.73, 95% CI = 3.22–10.21), but was only based on two studies. Exposure to lead, or pesticides was also linked to the occurrence of MS (ES = 1.63, 95% CI = 1.03–2.57 and ES = 1.22, 95% CI = 1.05–2.41, respectively), again, both based or largely driving by a single study. Similarly, having immigrated or experienced homelessness was associated with a higher risk of MS (ES = 2.60, 95% CI = 2.42–2.80 and ES = 4.60, 95% CI = 2.17–9.73, respectively), again, both based in single studies. Exposure to air pollution, radon gas, pets and having experienced a war situation were not associated with a risk of developing MS. The heterogeneity of the studies included in this subcategory was high (*I*^*2*^ = 97.41%). There was no publication bias among the studies (*p* = 0.70).

Overall, there was significant heterogeneity in all analyses, except for the analysis relating to BMI as a potential risk factor. Publication bias seemed present for BMI and vaccinations. It is also important to acknowledge that this review may have overlooked reverse causality and social determinants of health. For instance, individuals with early prodromal MS symptoms might modify their diet, or vaccination may be more common in populations with better access to healthcare. However, exploring the interplay between such variables, besides being complex, should be addressed in a separate original epidemiological study.

## Discussion

This systematic review with a meta-analysis stands as the most extensive assessment of environmental risk factors for MS. Our approach involved not only summarizing all the risk factors documented in original studies but also presenting updated evidence regarding the magnitude of their involvement in the etiopathogenesis of MS.

EBV serostatus has been extensively examined concerning their association with MS. Our review adds to the growing body of evidence underscoring the pivotal role of EBV in MS (ES = 1.96, 95% CI = 1.53–2.51) and aligns with findings suggesting that EBV appears to be a necessary cause (together with genetic susceptibility) [20, 21]. Recent studies have shed light on potential mechanisms underlying this connection. Notably, the EBV-specific T cell receptor repertoire appears broader in MS patients, suggesting an ongoing immune response to the EBV [22]. Additionally, antibodies recognizing an EBNA-1 epitope exhibit cross-reactivity with myelin basic protein in individuals with MS [23]. Consistent with these observations, some case reports suggest that antivirals targeting EBV may also show efficacy in alleviating MS symptoms [24]. However, despite the wealth of studies reviewed, several questions linger, including whether EBV serves as a trigger or driver of MS.

Other biological agents have been investigated as well. The pooled analyses revealed that previous infection by HHV-6 and VZV were associated with the risk of MS (ES = 2.84, 95% CI = 2.08–3.89 and ES = 1.27, 95% CI = 1.08–1.63). Our work is the first systematic review to provide evidence of these associations. Both viruses belong to the family of the human herpes virus, whose relationship with the nervous system has been extensively demonstrated. This group of viruses has a natural tropism for the nervous system, where they remain latent until there is another trigger for the exacerbation of the infection and activation of the immune system [25]. Herpes viruses are thought to reside in the sensory ganglia of the peripheral nervous system and share transynaptic dissemination pathways into the central nervous system (CNS) [26]. Once in the CNS, herpes virus may directly induce demyelination and/or modulate biological processes implicated in the remyelination, such as the expression of human endogenous retroviruses and the production of autoantibodies by molecular mimicry [27, 28]. Sotelo et al. suggested that VZV may play a specific role in MS relapses, as VZV DNA was found in 95% of MS patients during relapse and in 17% during remission [29]. This finding may support the hypothesis that this virus promotes disease activity. T lymphocytes isolated from patients with relapsing–remitting MS were induced to secret predominantly Th1 cytokines when stimulated by the VZV [30]. Lucas et al. found that the combination of EBNA IgG and HHV-6-DNA positivity had a stronger association with a first clinical diagnosis of CNS demyelination than either alone. The risk also seemed to be directly associated with the viral load [31]. Pietiläinen-Nicklén et al. showed that subjects with HHV-6 had significantly more oligoclonal bands in their cerebrospinal fluid, which is a hallmark of MS [32].

When examining vaccinations, we found that any vaccination could trigger an immune response potentially linked to MS onset [33–35]. Among specific vaccines, tetanus immunization was particularly associated with an increased risk of MS. Nevertheless, in some case additional studies may be needed to consolidate the evidence on the association of specific vaccines and MS, as in the case of the anti-tetanus vaccine, whose effect size comes from a single study whose primary object of investigation was not this vaccine specifically.

People who actively smoke were almost 50% more likely to be diagnosed with MS than non-smokers. The effect size found in this review could consolidate the evidence of smoking as a risk factor for MS, as indicated in the review carried out more than 10 years ago [36]. Supporting these observations, Manouchehrinia et al. estimated that 13% of the MS risk in Sweden is accounted for by smoking status [37]. Similar studies should be conducted in countries with varying smoking prevalence to better understand the true impact of smoking avoidance on MS risk. In parallel, some studies also demonstrated that smoking may accelerate the progression of MS [38] and increase the risk of conversion to clinically definite MS in patients with clinically isolated syndrome [39]. Notwithstanding, there is a lack of studies dedicated to exploring the underlying mechanism behind the relationship between smoking and MS [40]. In general, smoking is known to enhance the inflammatory response, increase the levels of proinflammatory cytokines and promote oxidative stress and specific epigenetic mechanisms that may the homeostasis of the central nervous system [41].

Obesity was not linked to MS in our study. There may be some reasons for the lack of a significant association. First, obesity is known to promote a systemic state of inflammation that is not specific to the cells of the nervous system [42]. Secondly, few studies provide detailed information on when BMI was measured and how it changed over time, which may introduce a major methodological flaw. In addition, neurological diseases that are clearly affected by obesity, such as stroke and dementia, usually manifest late, suggesting that the association may require a long period of exposure to be observed [43, 44]. Indeed, the lack of determination of the exact exposure period to a specific risk factor has been identified as one of the most common methodological flaws. In line with this possible explanation, there are many results that found a positive association between MS and obesity when the BMI was calculated in childhood [45]. Finally, the association may not be strong enough in real-world situations, where multiple confounding variables interact simultaneously.

Vitamin D levels were associated with MS: the lower the serum levels of serum 25-hydroxyvitamin D, the greater the risk (Hedges’s *g* = – 0.48, 95% CI = – 0.88, – 0.09). Vitamin D is recognized for its immunoregulatory effects on both innate and adaptive immunity, as documented by Gombash et al. [46]. In the context of the nervous system, vitamin D plays a role in regulating calcium-mediated neuronal excitotoxicity, decreasing oxidative stress, and promoting the synthesis of synaptic structural proteins, neurotrophic factors, and essential neurotransmitters [47]. Moreover, vitamin D assessment is considered analogous to the evaluation of factors such as latitude and sun exposure. This is primarily because these variables are closely linked to vitamin D levels, which is, in fact, the most plausible explanation for why some studies have previously found a relationship between latitude, sun exposure, and patients with MS [48, 49]. The proportion of people with MS classified as vitamin D deficient was also correlated with the proportion of cases of the disease (ES = 1.51 ng/mL, 95% CI = 1.10 ng/mL, – 2.06 ng/mL).

Many other environmental risk factors have been studied in MS. The vegetarian diet may play a role in the development of MS (ES = 5.73, 95% CI = 3.22–10.21), although the analysis was based on only two studies. The dietary pattern in general is known to have the potential to interact with the immune system [50], however, the impact of specific diets on the nervous system remains unclear. Indeed, we found a surprisingly association between MS risk and vegetarian diet. Preliminary evidence found the role of diet as a modulator of inflammatory neurological diseases [51]. A healthy diet could reduce the risk of MS [52]. Undoubtedly, this should guide the direction of future cohort and case–control studies. Environmental exposure to lead and pesticides were found to influence the MS risk (ES = 1.63, 95% CI = 1.03–2.57 and ES = 1.22, 95% CI = 1.05–1.41), although the evidence came from only a few studies. This association is supported by many publications that documented the multiple neurotoxic effects of heavy metals and chemical substances [53, 54]. Moreover, these observations are in line with the results of a recent meta-analyses that demonstrated the same associations in the occupational setting [55]. However, it is important to note that only one study investigated exposure to lead, and two studies examined exposure to pesticides. Immigration and homelessness experiences are adverse social experiences and as so, they may act as triggers to autoimmune diseases (ES = 2.60, 95%CI = 2.42–2.80 and ES = 4.60, 95% CI = 2.17–9.73) [56, 57]. Unfortunately, only one study investigated each of these exposures. However, they can be considered as situations that naturally make individuals susceptible to various environmental risk factors, especially biological ones, that may be involved in the risk of MS [58]. These findings guide the scope of future studies since the current evidence comes from few studies. Furthermore, for all these risk factors, the quantification and duration of exposure are still crucial to consolidate the knowledge about their real influence on the risk of developing MS, as these data have not been explored in any publication.

Respecting the rigor of the scientific method, it is important to acknowledge the inherent limitations of our study, although many of which are intrinsic to the study design of a systematic review with meta-analysis. Our effect size calculations could not account for confounding factors in the included studies, as this requires knowledge of the number of participants within each stratum of the confounder—a detail not publicly available. The inability to account for reporting bias in each study may introduce a significant limitation to the review. Another common non reported information was the duration of exposure which may be critical for some variables such as smoking. The quantitative analysis might also fail to fully capture the impact of methodological flaws identified individually. Furthermore, the lack of accurate consideration for time-varying factors, such as obesity—which likely has a time-dependent effect—could introduce another source of bias. Overall, substantial heterogeneity was observed, warranting caution in interpreting our results. Nevertheless, we addressed this issue by grouping studies into subgroups and excluding those with methodological uncertainties. Limiting the risk factors to be assessed during adulthood may influence the interpretation of the role of each variable. However, we decided to perform a separate study with childhood and adolescence risk factors as we understand they may act differently on the risk of MS, especially when MS also manifests in these periods (Pediatric-Onset MS). Finally, it is important to note that some effect sizes were derived from a limited number of studies, necessitating a more nuanced interpretation of the results.

## Conclusions

This comprehensive review furnishes a broad and detailed overview of the potential environmental risk factors associated with MS. Some exposures (EBV, smoking, vitamin D) are consistently associated with MS and merit further prospective or mechanistic study. Our review highlighted other environmental risk factors potentially linked to MS that should undoubtedly stimulate further studies. Overall, our findings hold notable implications for public health policies, clinical practices, and the focus of future research. MS is a multifactorial disease, but growing evidence confirms that environmental risk factors may play an important role in the pathogenesis of the disease. Ultimately, this study takes us strides closer to formulating effective strategies for the prevention and management of MS.

## Supplementary Information

Below is the link to the electronic supplementary material.

Supplementary file1 (DOCX 1263 KB)
