# Supplementary material for: Environmental risk factors for multiple sclerosis: a comprehensive systematic review and meta-analysis
Source: J Neurol. 2025 Jul 15;272(8):513. doi: 10.1007/s00415-025-13248-0 (PMC12263771; doi:10.1007/s00415-025-13248-0)
Supplement: Supplementary file 1 — Supplementary file1 (DOCX 1263 KB) [file 415_2025_13248_MOESM1_ESM.docx]

# Supplementary Table 1. Detailed search strategy.

| **PubMed** | (Employ* OR unemploy* OR occupation* OR work* OR environment* OR "job" OR "risk factor" OR "exposure" OR "aetiology" OR "physical" OR "chemical" OR "biological" OR "cause") AND ("multiple sclerosis") |
| --- | --- |
| **SciVerse Science Direct** | (“occupation” OR “work” OR “environment” OR "job" OR "risk factor" OR "exposure" OR "aetiology") AND (“multiple sclerosis”) |
| **Web of Science** | (Employ* OR unemploy* OR occupation* OR work* OR environment* OR "job" OR "risk factor" OR "exposure" OR "aetiology" OR "physical" OR "chemical" OR "biological" OR "cause") AND ("multiple sclerosis") |

# Supplementary Table 2. Detailed description of studies and participants included in the review.

| **Authors** | **Year** | **Country** | **N_CASES** | **N_CONTROLS** | **Age**  **Mean (SD)** | **Sex (%)** | **EDSS**  **MEAN (SD)** | **DISEASE DURATION**  **MEAN (SD)** | **DISEASE ONSET**  **MEAN (SD)** | **ENVIRONMENTAL EXPOSURE (S)** |
| --- | --- | --- | --- | --- | --- | --- | --- | --- | --- | --- |
| Abbasi et al. | 2017 | Iran | 660 | 421 |  | 558 (85.5) |  |  |  | Smoking, passive smoking, mercury exposure, pets exposure |
| Abdollahpour et al. | 2016 | Iran | 547 | 1057 | 30.5 (7.5) | 401 (73.3) |  |  |  | Smoking, passive smoking |
| Abdollahpour et al. | 2020 | Iran | 547 | 1057 | 31.3 (9.3) | 401 (73.3) |  |  |  | Smoking, passive smoking |
| Abdollahpour et al. | 2018 | Iran | 547 | 1057 | 31.3 (9.3) | 401 (73.3) |  |  |  | Measles, rubella, VZV, smoking |
| Abdollahpour et al. | 2018 | Iran | 547 | 1057 | 31.3 (9.3) | 401 (73.3) |  |  |  | Passive smoking, homelessness |
| Ahlgren et al. | 2009 | Sweden | 509 | 2067 |  |  |  |  |  | EBV, measles, mumps, rubella, VZV |
| Al-Afasy et al. | 2013 | Kuwait | 101 | 202 | 33.9 (9.1) | 56 (55.4) |  |  |  | War experience, smoking |
| Al-Shammri et al. | 2015 | Kuwait | 195 | 146 |  |  |  |  |  | BMI, smoking, war experience |
| Alonso et al. | 2011 | Iran | 394 | 394 | 30.9 (8.9) | 310 (78.7) |  | 6.2 (5.4) | 24.9 (8.9) | Measles, mumps, rubella, VZV, pets’ exposure |
| Alvarez-Lafuente et al. | 2006 | Spain | 57 | 57 | 34.5 (7.1) |  | 2.9 (1.2) | 7.7 (4.1) | 26.3 (6.5) | HHV-6 |
| Alvarez-Lafuente et al. | 2006 | Spain | 63 | 63 |  |  |  |  |  | HHV-6 |
| Alvarez-Lafuente et al. | 2002 | Spain | 103 | 46 |  | 75 (72.8) |  |  |  | EBV |
| Ammitzboll et al. | 1973 | Denmark | 92 | 72 |  |  |  |  |  | Measles |
| Andersen et al. | 1981 | Denmark | 81 | 243 |  |  |  |  |  | BCG vaccine |
| Ascherio et al. | 2001 | USA | 192 | 534 | 37.6 (NA) |  |  |  |  | Measles, smoking, HBV vaccine, measles vaccine, tetanus vaccine |
| Bansil et al. | 1997 | India | 56 | 91 | 34.1 (8.0) | 35 (62.5) |  |  | 28.0 (8.0) | Measles, mumps, VZV, pets’ exposure, BCG vaccine |
| Bettencourt et al. | 2017 | Portugal | 244 | 198 | 41.1 (11.3) | 151 (61.9) |  | 10.7 (8.7) |  | Vitamin D |
| Bistrom et al. | 2021 | Sweden | 670 | 670 |  | 563 (84.0) |  |  |  | EBV |
| Bjornevik et al | 2022 | USA | 955 | 1566 |  |  |  |  |  | EBV, CMV |
| Bjornevik et al | 2016 | Norway  Italy  Sweden | 953 | 1717 |  |  |  |  | 37.6 (10.2) | EBV, smoking |
| Boru et al. | 2020 | Turkey | 41 | 57779 | 39.8 (11.7) | 37 (90.2) | 3.8 (2.3) | 12.4 (8.9) |  | Air pollution |
| Briggs et al. | 2014 | USA | 1023 | 620 |  |  |  |  |  | EBV |
| Casetta et al. | 1994 | Italy | 104 | 150 | 45.6 (14.1) | 70 (67.3) |  |  |  | Pesticides, pets’ exposure |
| Cendrowski et al. | 1969 | Poland | 300 | 300 |  |  |  |  |  | Tuberculosis, VZV, BCG vaccine |
| Cendrowski et al. | 1973 | Poland | 80 | 990 |  |  |  |  |  | Measles |
| Cook et al. | 1977 | USA | 29 | 29 |  |  |  |  |  | Pets’ exposure |
| Corsenac et al. | 2022 | Canada | 1707 | 398856 |  |  |  |  |  | BCG vaccine |
| Csuka et al. | 2013 | Hungary | 135 | 345 |  | 76 (56.3) |  |  |  | EBV |
| Czarnowska et al. | 2018 | Poland | 141 | 44 | 41.6 (10.8) | 90 (63.8) | 2.2 (1.7) | 8.1 (7.2) | 33.4 (10.7) | EBV, CMV, HHV-6, HSV-1/2, VZV |
| de Jong et al. | 2019 | USA | 151 | 235 | 36.0 (4.5) | 151 (100) |  |  | 39.5 (8.9) | Pets’ exposure |
| de Villier e tal. | 2006 | South Africa | 49 | 39 |  |  |  |  |  | EBV, HHV-6 |
| Deeba et al. | 2019 | Cyprus | 133 | 101 | 48.3 (13.8) | 82 (61.6) |  |  |  | EBV |
| Dehghan et al. | 2018 | Iran | 120 | 360 | 30.9 (3.5) | 97 (80.8) |  |  |  | Vegetarian diet, smoking |
| Domínguez-Mozo et al. | 2021 | Spain | 191 | 79 |  |  |  |  |  | EBV, CMV, HHV-6 |
| Domínguez-Mozo et al. | 2022 | Spain | 325 | 295 |  | 217 (66.8) |  |  |  | EBV, smoking |
| Downham et al. | 2017 | UK | 9247 | 55033 |  |  |  |  |  | EBV |
| El-Muzaini et al. | 2020 | Kuwait | 110 | 110 | 34.8 (10.3) | 84 (76.4) |  |  | 27.3 (9.0) | BMI, war experience, smoking, Influenza vaccine, vitamin D |
| Eskandari et al. | 2015 | Iran | 45 | 45 | 30.3 (7.5) |  |  |  |  | Vitamin D |
| Fabis Pedrini et al. | 2015 | Australia | 550 | 299 | 47.7 (12.4) | 412 (74.9) |  |  | 35.4 (10.9) | *Helicobacter pylori* |
| Fathabadi et al. | 2020 | Iran | 45 | 100 |  |  |  |  |  | Radon gas exposure |
| Flodin et al, | 1988 | Sweden | 83 | 467 |  | 47 (56.6) |  |  |  | Pets’ exposure |
| Frutos-Alegria et al. | 2002 | Spain | 37 | 148 |  |  |  |  |  | Pets’ exposure |
| Gatto et al. | 2022 | Iceland | 214 | 27656 | 45.6 (11.1) |  |  |  |  | BMI, smoking |
| Ghadirian et al. | 2001 | Canada | 200 | 202 |  |  |  |  |  | Measles, mumps, rubella, smoking |
| Gianfrancesco et al. | 2014 | USA | 1235 | 697 |  | 986 (79.8) |  |  |  | BMI, EBV, smoking |
| Goulden et al. | 2016 | Italy  Norway  Canada | 655 | 1238 | 40.0 (9.4) | 425 (64.9) |  |  | 34.5 (9.3) | EBV, smoking |
| Grut et al. | 2021 | Sweden | 670 | 670 |  | 562 (83.9) |  |  |  | EBV, CMV, HHV-6 |
| Gudmundsdóttir et al. | 1979 | Iceland | 42 | 42 |  |  |  |  |  | CMV, measles, mumps, RSV, VZV |
| Gusev et al. | 1996 | Russia | 155 | 112 | 36.3 (9.8) | 96 (61.9) |  |  |  | Measles, mumps, rubella, VZV |
| Gustavsen et al. | 2014 | Norway | 756 | 1090 | 50.5 (12.7) | 391 (51.7) |  | 18.2 (11.6) | 32.3 (9.3) | EBV, smoking |
| Haahr et al. | 1995 | Denmark | 28 | 19739 |  |  |  |  |  | EBV |
| Halawani et al. | 2018 | Saudi Arabia | 80 | 160 |  | 52 (65.0) |  |  |  | Measles, VZV, smoking, passive smoking |
| Hawkes et al. | 2006 | UK | 277 | 1378 |  |  |  |  |  | HSV-1/2 |
| Hays et al. | 1992 | Canada | 63 | 63 |  |  |  |  |  | Mumps |
| Hedstrom e tal. | 2023 | Sweden | 6635 | 8880 |  | 4840 (72.9) |  |  | 35.2 (10.9) | EBV, air pollution |
| Hedstrom et al. | 2020 | Sweden | 7069 | 6632 |  |  |  |  |  | Vitamin D |
| Hedstrom et al. | 2009 | Sweden | 902 | 1855 |  |  |  |  |  | Smoking |
| Hedstrom et al. | 2014 | Sweden | 1311 | 1009 |  | 539 (41.1) |  |  |  | Passive smoking |
| Hedstrom et al. | 2016 | Sweden | 2455 | 5336 |  | 1760 (71.7) |  |  |  | Smoking, passive smoking |
| Hedstrom et al. | 2020 | Sweden | 6340 | 6219 |  | 4596 (72.5) |  |  |  | EBV, smoking |
| Hedstrom et al. | 2011 | Sweden | 695 | 1635 |  | 512 (73.7) |  |  |  | Smoking |
| Hernan et al. | 2000 | USA | 301 | 1416 |  |  |  |  |  | EBV, measles, mumps, rubella, VZV, pets’ exposure |
| Horng Kang et al. | 2011 | Taiwan | 53 | 1262200 |  |  |  |  |  | VZV |
| Ito et al. | 1975 | USA | 59 | 64 |  |  |  |  |  | HSV-1/2, VZV |
| Jafari et al. | 2009 | The Netherlands | 136 | 204 | 52.6 (11.4) | 86 (63.2) |  | 32.9 (9.5) |  | Smoking |
| Karampoor et al. | 2016 | Iran | 60 | 50 |  |  |  |  |  | EBV |
| Karampoor et al. | 2016 | Iran | 700 | 1000 | 41.0 (10.2) | 507 (72.4) | 4.0 (1.5) |  |  | Vitamin D |
| Karampoor et al. | 2017 | Iran | 800 | 1000 | 39.9 (12.6) | 600 (75.0) | 2.4 (1.8) |  |  | CMV |
| Keyvani et al. | 2020 | Iran | 263 | 263 |  | 140 (53.2) |  |  |  | HHV-6 |
| Khouy et al. | 2018 | Iran | 420 | 210 |  | 280 (66.7) |  |  |  | VZV |
| Kofahi et al. | 2020 | Jordania | 55 | 44 |  | 36 (65.4) |  |  |  | EBV, HHV-6, VZV |
| Koskderelioglu et al. | 2017 | Turkey | 115 | 60 | 41.2 (11.2) | 77 (66.9) |  |  |  | Toxoplasmosis |
| Kusumadewi et al. | 2018 | Indonesia | 29 | 33 |  |  |  |  |  | Vitamin D |
| Kuusisto et al. | 2004 | Finland | 19 | 15 |  | 14 (73.7) |  |  |  | Enterovirus |
| Langer-Gould e tal. | 2017 | USA | 519 | 571 |  | 242 (46.6) |  |  |  | EBV, CMV, smoking |
| Langer-Gould et al. | 2018 | USA | 247 | 267 | 39.7 (12.0) | 164 (66.4) |  |  |  | Smoking, vitamin D |
| Langer-Gould et al. | 2014 | USA | 780 | 3885 |  | 541 (69.3) |  |  |  | HBV vaccine, HPV vaccine |
| Levin et al. | 2010 | USA | 305 | 610 | 23.5 (5.4) | 103 (33.8) |  |  | 28.2 (6.0) | EBV |
| Lezhnyova et al. | 2022 | Russia | 124 | 70 | 33.7 (7.5) | 75 (60.5) | 2.8 (1.6) | 8.1 (5.8) |  | EBV, HHV-6, VZV |
| Li et al. | 2007 | Japan | 105 | 85 | 41.2 (13.2) | 81 (77.1) |  | 8.1 (7.5) | 32.9 (14.2) | *Helicobacter pylori* |
| Lonergan et al. | 2015 | Ireland | 632 | 480 |  |  |  |  |  | Vitamin D |
| Long et al. | 2013 | China | 42 | 27 |  | 21 (50.0) | 2.3 (1.6) | 6.2 (4.0) | 33.9 (10.6) | *Helicobacter pylori* |
| Lucas et al. | 2011 | Australia | 215 | 216 |  |  |  |  |  | EBV |
| Malli et al. | 2015 | India | 139 | 278 | 36.6 (12.0) | 92 (66.2) |  |  |  | *Helicobacter pylori,* measles, VZV, vegetarian diet |
| Mameli et al. | 2014 | Italy | 53 | 53 | 38.0 (11.0) | 28 (52.8) |  | 9.5 (10.0) | 31.0 (10.0) | EBV, *Mycobacterium avium* |
| Mansouri et al. | 2014 | Iran | 1217 | 787 |  | 921 (75.7) |  | 7.3 (4.2) |  | Smoking, measles vaccine, mumps vaccine, rubella vaccine, VZV vaccine |
| Maple et al. | 2019 | UK | 78 | 124 | 43.3 (1.3) | 57 (73.1) |  |  |  | EBV, CMV |
| Martin et al. | 1993 | UK | 214 | 160 |  |  |  |  |  | EBV |
| Martinez-Sobrepera et al. | 2001 | Cuba | 50 | 50 |  | 41 (82) |  |  |  | Measles |
| Mouhieddine et al. | 2015 | Lebanon | 249 | 230 |  |  | 2.0 (1.7) | 5.9 (7.1) |  | EBV |
| Munger et al. | 2011 | USA | 222 | 444 | 23.4 (5.5) |  |  |  | 28.4 (6.2) | EBV, smoking, vitamin D |
| Munger et al. | 2004 | USA | 129 | 258 |  |  |  |  |  | Chlamydia pneumoniae |
| Munger et al. | 2006 | USA | 148 | 296 |  |  |  |  |  | Vitamin D |
| Munger et al. | 2017 | Finland | 1092 | 2123 | 37.0 (7.1) | 1092 (100) |  |  | 37.0 (7.1) | Vitamin D |
| Myhr et al. | 1998 | Norway | 144 | 170 | 39.2 (NA) | 83 (57.6) |  | 6.9 (NA) |  | EBV, CMV, VZV, HSV-1/2 |
| Najafi et al. | 2016 | Iran | 82 | 89 | 36.9 (9.3) | 59 (71.9) |  |  | 7.9 (NA) | VZV |
| Napier et al. | 2016 | USA | 217 | 496 |  | 174 (80.2) |  |  |  | Lead exposure, mercury exposure |
| Nejati et al. | 2016 | Iran | 87 | 70 | 34.5 (8.8) |  |  |  |  | EBV |
| Niino et al. | 2015 | Japan | 70 | 40 | 45.8 (10.9) | 34 (48.6) | 3.6 (2.3) |  | 30.7 (11.2) | Vitamin D |
| Nordvelt et al. | 2004 | Norway | 87 | 22312 |  | 65 (74.7) |  | 12.8 (NA) | 33.0 (8.4) | Smoking |
| Pandit et al. | 2013 | India | 140 | 140 |  |  |  |  |  | EBV |
| Pandit et al. | 2013 | India | 110 | 108 |  |  |  |  |  | Vitamin D |
| Parron et al. | 2011 | Spain | 683 | 1832969 |  |  |  |  |  | Pesticides |
| Pekmezovic et al. | 2006 | Serbia | 210 | 210 | 33.6 (10.2) |  |  |  |  | Smoking |
| Pérez-Pérez et al. | 2019 | Spain | 53 | 58 | 43.4 (11.4) | 34 (64.1) | 2.0 (1.7) | 12.2 (8.1) | 31.1 (9.8) | Vitamin D |
| Perlejewski et al. | 2020 | Poland | 34 | 13 | 38.4 (13.8) | 20 (58.8) |  |  |  | EBV, enterovirus, HHV-6, VZV |
| Ramagopalan et al. | 2009 | Canada | 14362 | 7671 | 49.0 (9.6) | 10555 (73.5) |  |  |  | Measles, mumps, rubella, VZV, HBV vaccine, measles vaccine, mumps vaccine, rubella vaccine |
| Read et al. | 1982 | UK | 72 | 72 |  |  |  |  |  | Pets’ exposure |
| Rice et al. | 1986 | Canada | 201 | 29 |  |  |  |  |  | HTLV |
| Rodriguez-Violante et al. | 2009 | Mexico | 126 | 157 | 36.0 (10.0) | 87 (69.0) |  |  |  | VZV |
| Rotstein et al. | 2019 | Canada | 16513 | 8649958 |  | 11394 (69.0) |  |  | 37.1 (10.0) | Immigration |
| Sakoda et al. | 2019 | Japan | 103 | 124 | 43.6 (10.9) | 80 (77.7) | 2.8 (2.2) | 13.0 (8.6) |  | BMI |
| Scartezzini et al. | 2021 | Italy | 510 | 323617 |  | 354 (69.4) |  |  |  | Air pollution |
| Scazzone et al. | 2019 | Italy | 107 | 133 | 39.8 (9.9) | 26 (24.3) | 3.0 (2.2) | 11.6 (9.8) | 28.0 (7.9) | Vitamin D |
| Scheller et al. | 2015 | Denmark  Sweden | 4322 | 3978271 |  |  |  |  |  | HPV vaccine |
| Shaygannejad et al. | 2016 | Iran | 536 | 399 | 34.4 (9.2) |  | 2.6 (2.3) | 1.3 (2.7) |  | Measles |
| Shaygannejad et al. | 2010 | Iran | 50 | 50 | 36.6 (NA) | 42 (84) |  |  |  | Vitamin D |
| Siddiqui et al. | 2021 | Saudi Arabia | 82 | 82 |  | 50 (61.0) |  |  |  | BMI, pets’ exposure, smoking |
| Siejka et al. | 2016 | Australia | 136 | 272 | 43.5 (9.3) | 92 (67.6) | 3.5 (2.2) | 9.4 (7.5) | 34.6 (9.1) | EBV, smoking |
| Skalli et al. | 2018 | Morocco | 113 | 146 | 36.8 (10.8) |  | 3.5 (1.9) |  | 29.8 (10.0) | Vitamin D |
| Soilu-Hanninen et al. | 2005 | Finland | 40 | 40 | 36.5 (1.3) | 33 (82.5) | 1.5 (0.1) | 2.6 (0.5) |  | Vitamin D |
| Souberbielle et al. | 1990 | France | 230 | 230 | 30.7 (NA) | 143 (62.2) |  |  |  | EBV, diphtheria, HSV-1/2, malaria, measles, mumps, poliomyelitis, rubella, tuberculosis, VZV |
| Stascheit et al. | 2015 | Germany | 165 | 165 | 37.9 (11.9) | 108 (65.5) |  |  | 34.0 (10.2) | Toxoplasmosis, smoking |
| Sundqvist et al. | 2012 | Sweden | 792 | 1651 |  |  |  |  |  | EBV |
| Sundqvist et al. | 2013 | Sweden | 658 | 786 | 35.3 (9.9) | 469 (71.3) |  |  |  | CMV, smoking |
| Sundstrom et al. | 2008 | Sweden | 109 | 218 |  |  |  |  |  | Smoking |
| Survey et al. | 1983 | USA | 22 | 37 |  | 0 (0.0) |  |  |  | Pets’ exposure |
| van der Mei et al. | 2005 | Australia | 136 | 272 | 43.5 (9.3) | 92 (67.6) | 3.5 (2.2) | 9.4 (7.5) | 34.6 (9.1) | Vitamin D |
| Wagner et al. | 2000 | Germany | 107 | 163 | 37.8 (10.3) | 67 (62.6) |  | 7.7 (5.7) |  | EBV |
| Wesnes et al. | 2015 | Norway | 953 | 1717 |  | 790 (82.9) |  |  |  | BMI, vitamin D |
| Wutayd et al. | 2018 | Saudi Arabia | 307 | 307 | 32.9 (8.8) | 230 (74.9) |  |  |  | Measles, VZV, smoking |
| Zonzent et al. | 2003 | Italy | 140 | 131 | 42.1 (10.2) |  |  | 10.9 (7.5) |  | Measles, rubella, tuberculosis, VZV, pets’ exposure, smoking, BCG vaccine, measles vaccine, mumps vaccine, rubella vaccine, VZV vaccine |

# Supplementary Table 3. Critical appraisal of the studies included in the review.

| **Authors** | **Year** | **Q1** | **Q2** | **Q3** | **Q4** | **Q5** | **Q6** | **Q7** | **Q8** | **Q9** | **Q10** |
| --- | --- | --- | --- | --- | --- | --- | --- | --- | --- | --- | --- |
| Abbasi et al. | 2017 | YES | YES | YES | YES | YES | NO | NO | YES | UNCLEAR | YES |
| Abdollahpour et al. | 2018 | NO | NO | UNCLEAR | NO | YES | YES | YES | YES | UNCLEAR | YES |
| Abdollahpour et al. | 2018 | NO | NO | NO | NO | YES | YES | YES | YES | NO | YES |
| Abdollahpour et al. | 2020 | UNCLEAR | UNCLEAR | UNCLEAR | YES | YES | YES | YES | YES | YES | YES |
| Abdollahpour et al. | 2016 | NO | NO | NO | YES | YES | YES | YES | YES | YES | YES |
| Ahlgren et al. | 2009 | YES | YES | YES | NO | YES | YES | YES | YES | UNCLEAR | YES |
| Al-Afasy et al. | 2013 | YES | YES | YES | NO | YES | NO | NO | UNCLEAR | UNCLEAR | YES |
| Al-Shammri et al. | 2015 | NO | UNCLEAR | YES | NO | YES | YES | YES | YES | UNCLEAR | YES |
| Alonso et al. | 2011 | YES | UNCLEAR | UNCLEAR | NO | YES | YES | YES | UNCLEAR | UNCLEAR | YES |
| Alvarez-Lafuente et al. | 2006 | YES | YES | UNCLEAR | YES | YES | NO | NO | YES | UNCLEAR | YES |
| Alvarez-Lafuente et al. | 2002 | UNCLEAR | UNCLEAR | UNCLEAR | YES | YES | NO | NO | UNCLEAR | UNCLEAR | YES |
| Alvarez-Lafuente et al. | 2006 | UNCLEAR | UNCLEAR | UNCLEAR | YES | YES | NO | NO | UNCLEAR | UNCLEAR | YES |
| Ammitzboll et al. | 1973 | YES | UNCLEAR | YES | YES | YES | YES | YES | UNCLEAR | YES | YES |
| Andersen et al. | 1981 | UNCLEAR | UNCLEAR | YES | YES | YES | YES | NO | YES | YES | YES |
| Ascherio et al. | 2001 | YES | YES | YES | NO | YES | YES | YES | NO | UNCLEAR | YES |
| Bansil et al. | 1997 | UNCLEAR | YES | YES | NO | YES | NO | NO | YES | NO | YES |
| Bettencourt et al. | 2017 | NO | NO | UNCLEAR | YES | YES | YES | YES | YES | NO | YES |
| Bistrom et al. | 2021 | YES | YES | UNCLEAR | YES | YES | NO | NO | UNCLEAR | UNCLEAR | YES |
| Bjornevik et al | 2022 | NO | YES | NO | YES | YES | YES | YES | UNCLEAR | UNCLEAR | YES |
| Bjornevik et al | 2016 | YES | YES | YES | YES | YES | YES | YES | YES | YES | YES |
| Boru et al. | 2020 | UNCLEAR | UNCLEAR | YES | YES | YES | NO | NO | YES | YES | YES |
| Briggs et al. | 2014 | NO | YES | YES | YES | YES | YES | YES | YES | YES | YES |
| Casetta et al. | 1994 | UNCLEAR | UNCLEAR | YES | NO | YES | NO | NO | YES | UNCLEAR | YES |
| Cendrowski et al. | 1973 | UNCLEAR | UNCLEAR | UNCLEAR | YES | YES | NO | NO | UNCLEAR | UNCLEAR | YES |
| Cendrowski et al. | 1969 | NO | NO | NO | NO | YES | NO | NO | UNCLEAR | UNCLEAR | YES |
| Cook et al. | 1977 | UNCLEAR | YES | YES | YES | YES | NO | NO | YES | UNCLEAR | YES |
| Corsenac et al. | 2022 | UNCLEAR | UNCLEAR | YES | YES | YES | NO | NO | YES | YES | YES |
| Csuka et al. | 2013 | NO | NO | UNCLEAR | YES | YES | NO | NO | UNCLEAR | UNCLEAR | YES |
| Czarnowska et al. | 2018 | UNCLEAR | UNCLEAR | NO | YES | YES | NO | NO | YES | UNCLEAR | YES |
| de Jong et al. | 2019 | UNCLEAR | UNCLEAR | YES | YES | YES | YES | YES | YES | YES | YES |
| de Villier e tal. | 2006 | NO | NO | NO | YES | YES | NO | NO | UNCLEAR | UNCLEAR | YES |
| Deeba et al. | 2019 | YES | YES | UNCLEAR | YES | YES | NO | NO | YES | UNCLEAR | YES |
| Dehghan et al. | 2018 | YES | NO | UNCLEAR | NO | YES | NO | NO | YES | UNCLEAR | YES |
| Domínguez-Mozo et al. | 2022 | NO | NO | UNCLEAR | YES | YES | NO | NO | YES | UNCLEAR | YES |
| Domínguez-Mozo et al. | 2021 | YES | NO | UNCLEAR | YES | YES | NO | NO | YES | UNCLEAR | YES |
| Downham et al. | 2017 | NO | YES | YES | UNCLEAR | UNCLEAR | NO | NO | NO | NO | YES |
| El-Muzaini et al. | 2020 | YES | YES | YES | YES | YES | YES | YES | YES | UNCLEAR | YES |
| Eskandari et al. | 2015 | YES | YES | UNCLEAR | YES | YES | YES | YES | YES | NO | YES |
| Fabis Pedrini et al. | 2015 | YES | YES | YES | YES | YES | NO | NO | YES | UNCLEAR | YES |
| Fathabadi et al. | 2020 | NO | NO | YES | YES | YES | NO | NO | YES | UNCLEAR | YES |
| Flodin et al, | 1988 | NO | NO | NO | YES | YES | NO | NO | YES | UNCLEAR | YES |
| Frutos-Alegria et al. | 2002 | UNCLEAR | YES | YES | NO | YES | NO | NO | UNCLEAR | UNCLEAR | YES |
| Gatto et al. | 2022 | YES | UNCLEAR | YES | YES | YES | YES | YES | NO | UNCLEAR | YES |
| Ghadirian et al. | 2001 | YES | YES | YES | YES | YES | YES | YES | YES | YES | YES |
| Gianfrancesco et al. | 2014 | NO | NO | YES | UNCLEAR | YES | YES | YES | YES | YES | YES |
| Goulden et al. | 2016 | YES | YES | NO | YES | YES | YES | YES | YES | YES | YES |
| Grut et al. | 2021 | YES | YES | UNCLEAR | YES | YES | NO | NO | UNCLEAR | UNCLEAR | YES |
| Gudmundsdóttir et al. | 1979 | UNCLEAR | YES | NO | YES | YES | NO | NO | UNCLEAR | UNCLEAR | YES |
| Gusev et al. | 1996 | YES | YES | NO | NO | YES | YES | YES | YES | YES | YES |
| Gustavsen et al. | 2014 | NO | NO | NO | NO | YES | YES | YES | UNCLEAR | UNCLEAR | YES |
| Haahr et al. | 1995 | NO | NO | NO | YES | YES | NO | NO | NO | UNCLEAR | YES |
| Halawani et al. | 2018 | YES | YES | NO | YES | YES | YES | YES | YES | YES | YES |
| Hawkes et al. | 2006 | NO | NO | UNCLEAR | YES | YES | NO | NO | UNCLEAR | YES | YES |
| Hays et al. | 1992 | UNCLEAR | YES | UNCLEAR | NO | YES | NO | NO | UNCLEAR | YES | YES |
| Hedstrom e tal. | 2023 | YES | YES | YES | YES | YES | YES | YES | YES | NO | YES |
| Hedstrom et al. | 2020 | YES | YES | YES | YES | YES | YES | YES | YES | UNCLEAR | YES |
| Hedstrom et al. | 2020 | UNCLEAR | YES | NO | YES | YES | YES | YES | UNCLEAR | UNCLEAR | YES |
| Hedstrom et al. | 2014 | YES | YES | YES | NO | YES | YES | YES | YES | UNCLEAR | YES |
| Hedstrom et al. | 2011 | YES | YES | YES | YES | YES | YES | YES | YES | YES | YES |
| Hedstrom et al. | 2016 | YES | YES | UNCLEAR | YES | YES | YES | YES | YES | UNCLEAR | YES |
| Hedstrom et al. | 2009 | YES | YES | YES | YES | YES | YES | YES | YES | YES | YES |
| Hernan et al. | 2000 | YES | YES | YES | NO | YES | YES | YES | YES | YES | YES |
| Horng Kang et al. | 2011 | UNCLEAR | NO | YES | YES | YES | YES | YES | UNCLEAR | NO | YES |
| Ito et al. | 1975 | UNCLEAR | YES | NO | YES | YES | NO | NO | UNCLEAR | UNCLEAR | YES |
| Jafari et al. | 2009 | UNCLEAR | UNCLEAR | YES | YES | YES | YES | YES | YES | YES | YES |
| Karampoor et al. | 2017 | NO | NO | NO | YES | YES | NO | NO | YES | UNCLEAR | YES |
| Karampoor et al. | 2016 | YES | NO | UNCLEAR | YES | YES | NO | NO | YES | UNCLEAR | YES |
| Karampoor et al. | 2016 | YES | UNCLEAR | YES | UNCLEAR | YES | YES | YES | YES | UNCLEAR | YES |
| Keyvani et al. | 2020 | YES | YES | UNCLEAR | YES | YES | NO | NO | YES | UNCLEAR | YES |
| Khouy et al. | 2018 | UNCLEAR | UNCLEAR | UNCLEAR | YES | YES | NO | NO | UNCLEAR | UNCLEAR | YES |
| Kofahi et al. | 2020 | YES | NO | UNCLEAR | YES | YES | NO | NO | UNCLEAR | UNCLEAR | YES |
| Koskderelioglu et al. | 2017 | YES | YES | UNCLEAR | YES | YES | NO | NO | UNCLEAR | UNCLEAR | YES |
| Kusumadewi et al. | 2018 | YES | YES | NO | YES | YES | YES | NO | YES | UNCLEAR | NO |
| Kuusisto et al. | 2004 | YES | NO | YES | YES | YES | NO | NO | YES | UNCLEAR | YES |
| Langer-Gould e tal. | 2017 | YES | YES | YES | YES | YES | YES | YES | YES | UNCLEAR | YES |
| Langer-Gould et al. | 2018 | YES | YES | YES | YES | YES | YES | YES | YES | UNCLEAR | YES |
| Langer-Gould et al. | 2014 | YES | YES | YES | YES | YES | YES | YES | YES | UNCLEAR | YES |
| Levin et al. | 2010 | YES | YES | NO | YES | YES | YES | NO | YES | UNCLEAR | NO |
| Lezhnyova et al. | 2022 | YES | YES | UNCLEAR | YES | YES | NO | NO | YES | UNCLEAR | YES |
| Li et al. | 2007 | YES | UNCLEAR | UNCLEAR | YES | YES | NO | NO | YES | YES | YES |
| Lonergan et al. | 2015 | UNCLEAR | UNCLEAR | NO | YES | YES | NO | NO | YES | NO | YES |
| Long et al. | 2013 | YES | YES | UNCLEAR | UNCLEAR | UNCLEAR | NO | NO | YES | UNCLEAR | YES |
| Lucas et al. | 2011 | UNCLEAR | YES | YES | NO | YES | YES | YES | NO | YES | YES |
| Malli et al. | 2015 | YES | YES | UNCLEAR | NO | YES | YES | UNCLEAR | UNCLEAR | YES | YES |
| Mameli et al. | 2014 | YES | YES | NO | YES | YES | NO | NO | YES | UNCLEAR | YES |
| Mansouri et al. | 2014 | YES | YES | NO | NO | YES | YES | YES | YES | UNCLEAR | YES |
| Maple et al. | 2019 | YES | UNCLEAR | NO | YES | YES | NO | NO | YES | UNCLEAR | YES |
| Martin et al. | 1993 | NO | UNCLEAR | NO | UNCLEAR | UNCLEAR | NO | NO | UNCLEAR | UNCLEAR | YES |
| Martinez-Sobrepera et al. | 2001 | NO | NO | UNCLEAR | NO | YES | NO | NO | YES | UNCLEAR | YES |
| Mouhieddine et al. | 2015 | NO | NO | NO | YES | YES | YES | YES | UNCLEAR | UNCLEAR | YES |
| Munger et al. | 2004 | YES | YES | YES | YES | YES | YES | YES | YES | UNCLEAR | YES |
| Munger et al. | 2006 | YES | YES | YES | YES | YES | YES | YES | YES | YES | YES |
| Munger et al. | 2011 | YES | YES | YES | YES | YES | YES | YES | YES | UNCLEAR | YES |
| Munger et al. | 2017 | YES | YES | YES | YES | YES | YES | YES | YES | YES | YES |
| Myhr et al. | 1998 | UNCLEAR | YES | NO | YES | YES | YES | YES | UNCLEAR | UNCLEAR | YES |
| Najafi et al. | 2016 | NO | NO | NO | YES | YES | NO | NO | YES | UNCLEAR | YES |
| Napier et al. | 2016 | UNCLEAR | NO | UNCLEAR | NO | YES | NO | NO | YES | UNCLEAR | YES |
| Nejati et al. | 2016 | NO | NO | NO | YES | YES | NO | NO | UNCLEAR | UNCLEAR | YES |
| Niino et al. | 2015 | YES | YES | NO | YES | YES | NO | NO | YES | NO | YES |
| Nordvelt et al. | 2004 | UNCLEAR | UNCLEAR | UNCLEAR | YES | YES | NO | NO | NO | UNCLEAR | YES |
| Pandit et al. | 2013 | NO | YES | UNCLEAR | YES | YES | YES | YES | YES | UNCLEAR | YES |
| Pandit et al. | 2013 | YES | YES | YES | YES | YES | NO | NO | YES | NO | YES |
| Parron et al. | 2011 | NO | NO | UNCLEAR | NO | YES | NO | NO | UNCLEAR | UNCLEAR | YES |
| Pekmezovic et al. | 2006 | YES | YES | UNCLEAR | YES | YES | YES | YES | YES | YES | YES |
| Pérez-Pérez et al. | 2019 | YES | YES | UNCLEAR | YES | YES | YES | YES | YES | UNCLEAR | YES |
| Perlejewski et al. | 2020 | NO | NO | YES | YES | YES | NO | NO | YES | UNCLEAR | YES |
| Ramagopalan et al. | 2009 | NO | NO | NO | NO | YES | NO | NO | UNCLEAR | UNCLEAR | YES |
| Read et al. | 1982 | UNCLEAR | UNCLEAR | YES | YES | YES | NO | NO | YES | YES | YES |
| Rice et al. | 1986 | NO | UNCLEAR | NO | YES | YES | NO | NO | UNCLEAR | UNCLEAR | YES |
| Rodriguez-Violante et al. | 2009 | NO | NO | NO | YES | YES | NO | NO | YES | YES | YES |
| Rotstein et al. | 2019 | YES | YES | YES | YES | YES | YES | YES | UNCLEAR | YES | YES |
| Sakoda et al. | 2019 | YES | YES | NO | YES | YES | YES | YES | YES | YES | YES |
| Scartezzini et al. | 2021 | UNCLEAR | UNCLEAR | YES | YES | YES | NO | NO | YES | YES | YES |
| Scazzone et al. | 2019 | NO | NO | UNCLEAR | YES | YES | NO | NO | UNCLEAR | UNCLEAR | YES |
| Scheller et al. | 2015 | UNCLEAR | NO | YES | YES | YES | NO | NO | NO | YES | YES |
| Shaygannejad et al. | 2010 | UNCLEAR | YES | NO | YES | YES | NO | NO | YES | UNCLEAR | YES |
| Shaygannejad et al. | 2016 | NO | NO | NO | UNCLEAR | YES | NO | NO | YES | UNCLEAR | YES |
| Siddiqui et al. | 2021 | NO | NO | NO | NO | YES | NO | NO | YES | UNCLEAR | YES |
| Siejka et al. | 2016 | YES | YES | YES | YES | YES | YES | YES | YES | YES | YES |
| Skalli et al. | 2018 | UNCLEAR | YES | NO | YES | YES | YES | YES | YES | UNCLEAR | YES |
| Soilu-Hanninen et al. | 2005 | UNCLEAR | UNCLEAR | NO | YES | YES | YES | YES | YES | UNCLEAR | YES |
| Souberbielle et al. | 1990 | UNCLEAR | UNCLEAR | NO | NO | YES | NO | NO | YES | UNCLEAR | YES |
| Stascheit et al. | 2015 | YES | YES | NO | YES | YES | NO | NO | YES | UNCLEAR | YES |
| Sundqvist et al. | 2013 | YES | NO | YES | YES | YES | YES | YES | YES | UNCLEAR | YES |
| Sundqvist et al. | 2012 | NO | YES | NO | YES | YES | NO | NO | YES | UNCLEAR | YES |
| Sundstrom et al. | 2008 | UNCLEAR | UNCLEAR | UNCLEAR | YES | YES | NO | NO | UNCLEAR | UNCLEAR | YES |
| Survey et al. | 1983 | UNCLEAR | YES | NO | YES | YES | NO | NO | UNCLEAR | YES | YES |
| van der Mei et al. | 2005 | YES | YES | YES | YES | YES | YES | YES | YES | NO | YES |
| Wagner et al. | 2000 | NO | YES | NO | YES | YES | NO | NO | YES | UNCLEAR | YES |
| Wesnes et al. | 2015 | NO | YES | UNCLEAR | YES | YES | YES | YES | UNCLEAR | YES | YES |
| Wutayd et al. | 2018 | YES | YES | YES | NO | YES | YES | YES | YES | UNCLEAR | YES |
| Zonzent et al. | 2003 | UNCLEAR | YES | YES | NO | YES | YES | YES | YES | UNCLEAR | YES |

# Supplementary Figure 1. Meta-analysis of the association between vaccines and MS.

# Supplementary Figure 2. Meta-analysis of the association between BMI and MS.

# Supplementary Figure 3. Meta-analysis of the association between vitamin D levels and MS.

Supplementary Table 4

| **Section and Topic** | **Item #** | **Checklist item** | **Location where item is reported** |
| --- | --- | --- | --- |
| **TITLE** | | |  |
| Title | 1 | Identify the report as a systematic review. | p. 1 |
| **ABSTRACT** | | |  |
| Abstract | 2 | See the PRISMA 2020 for Abstracts checklist. | p. 3 |
| **INTRODUCTION** | | |  |
| Rationale | 3 | Describe the rationale for the review in the context of existing knowledge. | pp. 5, 6 |
| Objectives | 4 | Provide an explicit statement of the objective(s) or question(s) the review addresses. | p. 6 |
| **METHODS** | | |  |
| Eligibility criteria | 5 | Specify the inclusion and exclusion criteria for the review and how studies were grouped for the syntheses. | pp. 7, 8 |
| Information sources | 6 | Specify all databases, registers, websites, organisations, reference lists and other sources searched or consulted to identify studies. Specify the date when each source was last searched or consulted. | p. 7, Figure 1 |
| Search strategy | 7 | Present the full search strategies for all databases, registers and websites, including any filters and limits used. | eTable 1 |
| Selection process | 8 | Specify the methods used to decide whether a study met the inclusion criteria of the review, including how many reviewers screened each record and each report retrieved, whether they worked independently, and if applicable, details of automation tools used in the process. | p. 7 |
| Data collection process | 9 | Specify the methods used to collect data from reports, including how many reviewers collected data from each report, whether they worked independently, any processes for obtaining or confirming data from study investigators, and if applicable, details of automation tools used in the process. | p. 8 |
| Data items | 10a | List and define all outcomes for which data were sought. Specify whether all results that were compatible with each outcome domain in each study were sought (e.g. for all measures, time points, analyses), and if not, the methods used to decide which results to collect. | p. 8 |
|  | 10b | List and define all other variables for which data were sought (e.g. participant and intervention characteristics, funding sources). Describe any assumptions made about any missing or unclear information. | p. 8 |
| Study risk of bias assessment | 11 | Specify the methods used to assess risk of bias in the included studies, including details of the tool(s) used, how many reviewers assessed each study and whether they worked independently, and if applicable, details of automation tools used in the process. | p. 9 |
| Effect measures | 12 | Specify for each outcome the effect measure(s) (e.g. risk ratio, mean difference) used in the synthesis or presentation of results. | p. 9 |
| Synthesis methods | 13a | Describe the processes used to decide which studies were eligible for each synthesis (e.g. tabulating the study intervention characteristics and comparing against the planned groups for each synthesis (item #5)). | pp. 8, 9 |
|  | 13b | Describe any methods required to prepare the data for presentation or synthesis, such as handling of missing summary statistics, or data conversions. | pp. 8, 9 |
|  | 13c | Describe any methods used to tabulate or visually display results of individual studies and syntheses. | pp. 8, 9 |
|  | 13d | Describe any methods used to synthesize results and provide a rationale for the choice(s). If meta-analysis was performed, describe the model(s), method(s) to identify the presence and extent of statistical heterogeneity, and software package(s) used. | p. 9 |
|  | 13e | Describe any methods used to explore possible causes of heterogeneity among study results (e.g. subgroup analysis, meta-regression). | p. 9 |
|  | 13f | Describe any sensitivity analyses conducted to assess robustness of the synthesized results. | p. 9 |
| Reporting bias assessment | 14 | Describe any methods used to assess risk of bias due to missing results in a synthesis (arising from reporting biases). | p. 9 |
| Certainty assessment | 15 | Describe any methods used to assess certainty (or confidence) in the body of evidence for an outcome. | pp.9, 10 |
| **RESULTS** | | |  |
| Study selection | 16a | Describe the results of the search and selection process, from the number of records identified in the search to the number of studies included in the review, ideally using a flow diagram. | p. 10 and Figure 1 |
|  | 16b | Cite studies that might appear to meet the inclusion criteria, but which were excluded, and explain why they were excluded. | p. 10 and Figure 1 |
| Study characteristics | 17 | Cite each included study and present its characteristics. | eTable 2 |
| Risk of bias in studies | 18 | Present assessments of risk of bias for each included study. | p. 10 and eTable 3 |
| Results of individual studies | 19 | For all outcomes, present, for each study: (a) summary statistics for each group (where appropriate) and (b) an effect estimate and its precision (e.g. confidence/credible interval), ideally using structured tables or plots. | Figures 2 – 6 and eFigures 1 – 3 |
| Results of syntheses | 20a | For each synthesis, briefly summarise the characteristics and risk of bias among contributing studies. | p. 10 |
|  | 20b | Present results of all statistical syntheses conducted. If meta-analysis was done, present for each the summary estimate and its precision (e.g. confidence/credible interval) and measures of statistical heterogeneity. If comparing groups, describe the direction of the effect. | pp. 10 – 12, Figures 2 – 6 and eFigures 1 – 3 |
|  | 20c | Present results of all investigations of possible causes of heterogeneity among study results. | pp. 10 – 12 |
|  | 20d | Present results of all sensitivity analyses conducted to assess the robustness of the synthesized results. | pp. 10 – 12 |
| Reporting biases | 21 | Present assessments of risk of bias due to missing results (arising from reporting biases) for each synthesis assessed. | pp. 10 – 12 |
| Certainty of evidence | 22 | Present assessments of certainty (or confidence) in the body of evidence for each outcome assessed. | pp. 10 – 12, Figures 2 – 6 and eFigures 1 – 3 |
| **DISCUSSION** | | |  |
| Discussion | 23a | Provide a general interpretation of the results in the context of other evidence. | p. 12 |
|  | 23b | Discuss any limitations of the evidence included in the review. | pp. 12 – 16 |
|  | 23c | Discuss any limitations of the review processes used. | p. 16 |
|  | 23d | Discuss implications of the results for practice, policy, and future research. | p. 12 |
| **OTHER INFORMATION** | | |  |
| Registration and protocol | 24a | Provide registration information for the review, including register name and registration number, or state that the review was not registered. | p. 7 |
|  | 24b | Indicate where the review protocol can be accessed, or state that a protocol was not prepared. | p. 7 |
|  | 24c | Describe and explain any amendments to information provided at registration or in the protocol. | NA |
| Support | 25 | Describe sources of financial or non-financial support for the review, and the role of the funders or sponsors in the review. | p. 2 |
| Competing interests | 26 | Declare any competing interests of review authors. | p. 2 |
| Availability of data, code and other materials | 27 | Report which of the following are publicly available and where they can be found: template data collection forms; data extracted from included studies; data used for all analyses; analytic code; any other materials used in the review. | NA |
